# Feasibility of collecting computer-facilitated patient-reported tobacco use, interest, and preferences for smoking cessation in an outpatient thoracic surgery and oncology setting

**DOI:** 10.18332/tid/150335

**Published:** 2022-07-04

**Authors:** Manan M. Nayak, Emanuele Mazzola, Michael T. Jaklitsch, Jeremy E. Drehmer, Emara Nabi-Burza, Raphael Bueno, Jonathan P. Winickoff, Mary E. Cooley

**Affiliations:** 1 The Phyllis F. Cantor Center Research in Nursing and Patient Care Services, Dana-Farber Cancer Institute, Boston, United States; 2 Department of Psychosocial Oncology and Palliative Care, Dana-Farber Cancer Institute, Boston, United States; 3 Department of Data Science, Dana-Farber Cancer Institute, Boston, United States; 4 Division of Thoracic and Cardiac Surgery, Brigham and Women’s Hospital, Boston, United States; 5 Division of General Academic Pediatrics, Massachusetts General Hospital for Children, Boston, United States; 6 Tobacco Research and Treatment Center, Massachusetts General Hospital, Boston, United States

**Keywords:** electronic collection of tobacco use data, smoking cessation treatment, thoracic surgery, household tobacco exposure, brief smoking cessation interventions

## Abstract

**INTRODUCTION:**

Effective strategies are needed to facilitate collection of tobacco use information and integrate smoking cessation treatment into the routine care of all high-risk patient populations to improve clinical outcomes. The objective of this study was to establish the feasibility of collecting computer-facilitated patient-reported tobacco use, identify patient interest and preferences for smoking cessation in an outpatient thoracic surgery and oncology setting with higher prevalence of tobacco use than the general population.

**METHODS:**

A brief patient-administered tobacco screening survey was handed out on an iPad in the waiting room of a thoracic surgery and oncology practice setting to sequential patients with varying diagnoses. Tobacco use, household exposure to tobacco, and interest and preferences for smoking cessation treatment were recorded. Descriptive statistics and Pearson’s chi-squared test were used for analysis.

**RESULTS:**

Of the 599 surveys administered, 594 (99%) were completed. Self-reported smoking status included 36.4% (n=218) never smokers, 53.3% (n=319) former smokers, and 10.4% (n=62) current smokers. Among current smokers, 45.2% (n=28) were interested in receiving smoking cessation treatment. Preferences for treatment included: 21.4% (n=6) who wanted Quitline only, 25% (n=7) medication alone, and 53.6% (n=15) combined Quitline plus medication. Current smokers (55.7%, n=34) were more likely to live in households with tobacco exposure compared to those with former (11.4%, n=36) or never smokers (8.3%, n=18) (p<0.0001).

**CONCLUSIONS:**

Implementing a computer-facilitated system to screen for current smoking and provide smoking cessation services was feasible in the outpatient thoracic surgery and oncology setting. Almost half of the smokers indicated an interest in receipt of smoking cessation treatment. Household exposure was more frequent among current smokers, therefore routine screening for secondhand smoke exposure from other household members is an important consideration in developing smoking cessation treatment plans to mitigate health risks among vulnerable patient populations.

## INTRODUCTION

Smoking cessation is essential for patients undergoing thoracic surgery, as continued smoking before surgery is associated with higher rates of pulmonary and wound healing complications^1^. Smoking cessation is important even after lung cancer surgery since continued smoking is associated with recurrent cancer and decreased survival^2^. Effective treatments for smoking cessation are available but are often not integrated into routine care. Combined use of medication and behavioral counseling are considered ‘best practice’ and can double cessation rates^3^. A barrier to implementing treatment is the lack of a system-level approach to identify and offer smoking cessation services to all smokers.

Brief system-level interventions are effective in providing smoking cessation treatments and require minimal resources and expertise to implement within practice settings. One model that has been implemented extensively uses three steps^4^: 1) Ask about tobacco use, 2) Assist smokers by offering treatment programs and medications, and 3) Refer smokers to smoking cessation programs (i.e. Quitlines).

Although rates of documenting tobacco use are high in the outpatient setting, rates for assisting or referring patients for treatment are much lower^5^. In a survey of thoracic surgeons, only 9% provided nicotine replacement treatment (NRT) and referred smokers for counseling^6^. It is essential to employ strategies to gather tobacco use information that can be acted upon at the point-of-care to deliver smoking cessation treatment. Previous studies have increased the delivery of smoking cessation treatments by using iPads within primary care offices to screen people for tobacco use and identify preferences for cessation treatment, however, to our knowledge, no previous studies have assessed the implementation of computer-facilitated systems to collect tobacco use data within thoracic surgery and oncology settings^7,8^.

The objective of this study was to establish the feasibility of collecting computer-facilitated patient-reported tobacco use, and identify patient interest and preferences for smoking cessation in an outpatient thoracic surgery and oncology setting. This setting was chosen because the prevalence of tobacco use tends to be higher compared to the general population and implementation of smoking cessation treatment is essential to enhance clinical outcomes among those undergoing surgical treatment for thoracic diseases and improves efficacy of cancer treatment and survival among those diagnosed with lung cancer. Feasibility was defined as 80% completion of the tobacco survey questions collected through the iPad. The specific aims of the study were to: 1) identify the completion rate of a patient-administered tobacco survey; 2) describe the smoking status and preferences for smoking cessation treatment among patients in an outpatient thoracic surgery and oncology clinic; and 3) examine differences in household tobacco exposure by patient smoking status, which can influence the uptake of cessation services. We hypothesized that household tobacco exposure would be higher among current smokers compared to former or never smokers.

## METHODS

### Study design

This was a cross-sectional study that administered a tobacco survey using an iPad to sequential patients in a large academic outpatient setting with mixed diagnoses, including smoking and non-smoking related diagnoses, that was located in an urban northeastern city. Dana-Farber/Harvard Cancer Center Institutional Review Board reviewed and approved this study.

### Procedures

The research staff met with clinicians and front-desk staff to discuss smoking cessation treatment, summarized by the 3-step approach of Ask, Assist, and Refer^4^. This approach provided an easy way for clinical staff not familiar with smoking cessation treatment delivery to integrate evidence-based interventions into their practice. The screening survey was designed to be integrated as part of routine care, minimize interruption to the clinic workflow, and did not record the name of the patient.

The front-desk staff handed an iPad to patients during their check-in for clinic appointments to initiate the tobacco survey. Patients entered their data regarding tobacco use and exposure, which enabled further evaluation of patient interest and preferences for smoking cessation treatment among current smokers^8^. After survey completion, a message alerted the patient to return the iPad to the front desk. The iPad displayed a message for the front-desk staff indicating patient preferences regarding smoking cessation treatment. The front desk staff confirmed the patient’s name and manually flagged medical charts of individuals reporting current smoking, alerting the clinician of patient preferences for treatment, including those not interested in receiving smoking cessation services. At the time of the visit, clinicians provided a referral to Quitline, and/or a prescription for over-the-counter nicotine replacement therapy (NRT).

### Measures

*Tobacco use*

A standardized questionnaire was used to collect patient-reported tobacco use data^9^. Current smoking was defined as smoking within the past 30 days.

*Household exposure*

Household exposure to tobacco was defined as secondhand smoke exposure from any members of the household.

*Interest in cessation services*

Data were collected on patient interest, and preferences for treatment, including medications and behavioral counseling through Quitline.

*Appointment information*

Participants were asked to select the clinician they were seeing to facilitate communication of patient interest and receipt of smoking cessation treatment.

*Survey completion*

A single dichotomous question assessed if the survey was completed within 6 months. Individuals who previously completed the survey in the last 6 months completed a shorter version of the survey.

### Data collection

The screening survey was administered through a secure web-based application designed for research studies and took between 1 and 5 minutes to complete, with a minimum of 4 and a maximum of 13 questions. The survey utilized branching logic based on four measures that determined the survey length: lifetime tobacco use, current smoking status, household tobacco exposure, and survey completion within 6 months.

### Data analysis

Data were summarized using frequencies and percentages for tobacco-related data. Association between smoking status and household exposure was tested through Pearson’s chi-squared test.

## RESULTS

*Completion rate for patient-reported tobacco survey*

A total of 599 surveys were administered, and the completion rate was 99.0% (n=594) for the required fields (i.e. smoking at least 100 cigarettes in their lifetime, smoking within the last 30 days, household tobacco exposure).

*Smoking status and preferences for tobacco treatment*

Among the 599 participants with an appointment at the thoracic surgery and oncology clinic, 36.4% (n=218) self-reported being never smokers, 53.3% (n=319) were former smokers, and 10.4% (n=62) identified as current smokers. Among the 319 former smokers, 55.2% (n=176) reported quitting smoking >15 years ago, 16.3% (n=52) quit 6–15 years ago, 12.9% (n=41) quit between 2–5 years ago, and 13.2% (n=42) quit within the past year.

Among the 62 current smokers, 45.2% (n=28) were interested in receiving some type of smoking cessation treatment. Preferences for treatment included the following: 21.4% (n=6) wanted Quitline only, 25% (n=7) medication only (i.e. NRT or bupropion), and 53.6% (n=15) a combination of Quitline plus medication.

*Differences in household tobacco exposure by patient smoking status*

Household tobacco exposure was 14.8% (n=88) among all patients. We examined rates for household exposure by patient smoking status and found that current smokers (55.7%, n=34) were more likely to have experienced household tobacco exposure compared to former (11.4%, n=36) or never smokers (8.3%, n=18) (χ^2^=98.3013, p<0.0001).

## DISCUSSION

Computer-facilitated collection of patient-reported tobacco use, interest, and preferences for smoking cessation survey was feasible in an outpatient thoracic surgery and oncology setting. Although routine collection of patient-reported outcome (PRO) data is a priority to enhance the quality of care, integration into busy clinical settings can be a challenge. Heiden et al.^10^ implemented PRO data collection into a thoracic surgery clinic and identified key drivers for successfully integrating PROs; having engaged staff and patients, adequate technological capacity, and adequate time for survey completion. Like Heiden et al.^10^, we limited the number of survey questions, so they were completed quickly and efficiently before a clinic visit and did not interfere with the clinic workflow.

Delivery of smoking cessation interventions within thoracic surgery and oncology settings is essential to improve patient outcomes. The results from our study suggest that a system-level approach automating the collection of tobacco use and identifying those interested in treatment has the potential to improve processes of care. Approximately 45% of the patients who were smoking indicated an interest in some form of treatment. This level of interest in receipt of smoking cessation treatment is similar to a study by Mustoe et al.^11^ that examined patient engagement with smoking cessation treatment provided by the Quitline in a thoracic surgery setting.

Household tobacco exposure was 14% overall, however, we found significantly higher tobacco exposure (55.7%) among current smokers. These results underscore the importance of smoke-free homes as effective strategies to complement smoking cessation interventions. Prior studies have found household bans on smoking were much lower among current smokers than non-smokers^12^. Blok et al.^13^ examined the effect of smoking within social networks on smoking cessation and relapse among adults and found that household members with smokers were less likely to quit smoking and more likely to relapse. Home smoking bans among treatment-seeking adults are a potentially effective way to enhance cessation rates among all household members. A prior study found that the 30-day cessation rates were highest among Quitline callers who implemented a complete household smoking ban (51%) compared to those who implemented a partial ban (27%) or no ban (14%)^14^. In the thoracic surgery and oncology context, minimizing exposure to tobacco smoke is especially important to optimize treatment outcomes for the patient, and setting home and car smoking rules might also facilitate cessation among household members. Overall, a greater number of household smokers were found among non-smoking and former smoking patients, indicating the importance of screening all high-risk patients for household tobacco use exposure. Clinical encounters, offering evidence-based treatment to smokers in the patients’ household, have been successful in other settings^15^ and is a model that can be tested in thoracic and oncologic setting.

### Limitations

This study aimed to establish the feasibility of computer-facilitated collection of a patient-reported tobacco survey. We collected anonymous tobacco use data on the iPads. As a result, only the clinic staff had access to patient information rather than the research staff, limiting our ability to identify the demographic characteristics of those completing the survey. The front desk staff did not keep a record of patients who declined to complete the survey, therefore the actual number of current smokers in the clinic may be greater than we identified.

## CONCLUSIONS

Implementing a computer-facilitated system to screen for current smoking and provide smoking cessation services was feasible in the outpatient thoracic surgery and oncology setting. We found almost half of the smokers indicated an interest in receipt of smoking cessation treatment.

Household exposure was more frequent among current smokers, therefore routine screening for secondhand smoke exposure from other household members is an important consideration in developing smoking cessation treatment plans to mitigate health risks and protect vulnerable patient populations.

## Data Availability

The data supporting this research are available from the authors on reasonable request.
